# Application of the estimand framework for an emulated trial using reference based multiple imputation to investigate informative censoring

**DOI:** 10.1186/s12874-024-02364-6

**Published:** 2024-10-18

**Authors:** A. Atkinson, M. Zwahlen, S. De Wit, H. Furrer, J. R. Carpenter

**Affiliations:** 1 Division of Infectious Diseases, Washington University in St. Louis School of Medicine, St. Louis, MO USA; 2 Department of Medical Statistics, London School of Hygiene and Tropical Medicine, London, UK; 3 Institute of Social and Preventive Medicine, University of Bern, Bern, Switzerland; 4 Department of Infectious Diseases, Saint Pierre University Hospital, Université Libre de Bruxelles, Brussels, Belgium; 5 MRC Clinical Trials Unit, University College London, London, UK; 6 Department of Infectious Diseases, Bern University Hospital, Inselspital, Bern, Switzerland

**Keywords:** Estimand framework, Informative censoring, Multiple imputation, Sensitivity analysis, Trial emulation

## Abstract

**Background:**

The ICH E9 (R1) addendum on Estimands and Sensitivity analysis in Clinical trials proposes a framework for the design and analysis of clinical trials aimed at improving clarity around the definition of the targeted treatment effect (the *estimand*) of a study.

**Methods:**

We adopt the estimand framework in the context of a study using “trial emulation” to estimate the risk of pneumocystis pneumonia, an opportunistic disease contracted by people living with HIV and AIDS having a weakened immune system, when considering two antibiotic treatment regimes for stopping antibiotic prophylaxis treatment against this disease. A “while on treatment” strategy has been implemented for post-randomisation (intercurrent) events. We then perform a sensitivity analysis using *reference based multiple imputation* to model a scenario in which patients lost to follow-up stop taking prophylaxis.

**Results:**

The primary analysis indicated a protective effect for the new regime which used viral suppression as prophylaxis stopping criteria (hazard ratio (HR) 0.78, 95% confidence interval [0.69, 0.89], *p* < 0.001). For the sensitivity analysis, when we apply the “jump to off prophylaxis” approach, the hazard ratio is almost the same compared to that from the primary analysis (HR 0.80 [0.69, 0.95], *p* = 0.009). The sensitivity analysis confirmed that the new regime exhibits a clear improvement over the existing guidelines for PcP prophylaxis when those lost to follow-up “jump to off prophylaxis”.

**Conclusions:**

Our application using reference based multiple imputation demonstrates the method’s flexibility and simplicity for sensitivity analyses in the context of the estimand framework for (emulated) trials.

**Supplementary Information:**

The online version contains supplementary material available at 10.1186/s12874-024-02364-6.

## Background

The ICH E9 (R1) addendum on Estimands and Sensitivity analysis in Clinical trials proposes a framework for the design and analysis of clinical trials aimed at improving clarity around the definition of the targeted treatment effect (the estimand) of a study, and formally separating this from the analysis approach [1, 2]. An estimand is defined in terms of five components: the target population, treatment regimens to be compared, the outcome definition, the population-level summary and the strategies for handling post-randomisation events, known hereafter as “intercurrent events” (ICEs). The ICH E9 addendum also proposes changes in the way we consider missing data in the context of the estimand framework. In our case study, we present an example in which a *while on treatment* strategy has been implemented for the ICE “non-compliance with treatment regime” in the primary analysis. This strategy aims to evaluate the effect of the treatments being compared before the ICE has occurred. (In contrast to a “treatment policy” strategy for ICEs in which the occurrence of an intercurrent event is taken to be part of the treatment).

Since we do not know what happens to patients after they are lost to follow-up (LTFU), we use a controlled multiple imputation (MI) approach to then perform a *sensitivity analysis* to investigate a plausible scenario which is different from the standard assumptions of the primary analysis [3]. As Rehal et al. recently reported “a key aspect of the estimand framework is that missing data is a problem *for the estimator not the estimand*….missing data is not viewed as an [ICE per se] but there may also be missing data as a consequence of an [ICE]” [italics added] [4].

Of course, the estimand framework was developed with randomised controlled trials in mind. Recent advances in the analysis of observational data using causal methods, and specifically, the “emulation” of trials using observational data, means that the estimand framework can also be adopted here. In our application, we adopt this framework in the context of a trial emulation to estimate the risk of primary pneumocystis pneumonia (PcP), an opportunistic disease contracted by people living with HIV and AIDS (PLWHA), when considering two treatment regimes for stopping antibiotic prophylaxis treatment against this disease. PLWHA at risk for PcP are generally prescribed antiretroviral therapy (ART) in order to suppress plasma viral load, along with prophylactic treatments. Once viral load is managed, stopping prophylactic treatments as soon as possible is preferred since, notwithstanding increased pill burden, they can cause adverse events and increase the risk of antibacterial resistance.

## Methods

### Case Study: withholding primary pneumocystis pneumonia (PcP) prophylaxis in virologically suppressed PLWHA

The Collaboration of Observational HIV Epidemiological Research Europe (COHERE) in EuroCoord was a project-based collaboration which comprised 40 adult, paediatric, and mother/child HIV cohorts across Europe [5]. Due to the low incidence of PcP for patients on combined AntiRetroviral Therapy (cART), a randomised trial would be prohibitive, both in terms of time and cost. An emulated trial using observational data from COHERE offers a viable alternative to estimate the risk of a proposed new treatment regime [6, 7], and this approach was recently implemented [8].

The hypothetical “target” trial from the original study was a two arm, open label, 5-year study comparing the risk of two regimes for stopping PcP prophylaxis in terms of a composite primary endpoint of PcP diagnosis or all-cause death. HIV infected individuals were eligible to enter the hypothetical target trial if (i) they began follow-up in their cohort after 1st June 1998, (ii) they started cART on or after this date, (iii) were 16 years or older, (iv) had no history of previous PCP, and finally, (v) were taking PcP prophylaxis in line with existing recommendations, (i.e. they were taking prophylaxis if they had a CD4 count of less than < 200 cells/µL) (refer to Table S1). If eligible, patients were randomized to one of the two PcP prophylaxis stopping regimes:

- Regime 1 (*current recommendation*): Continue taking PcP prophylaxis if CD4 < 200 cells/µL, and stop if CD4 increases from < 200 to > 200 cells/µL for > 3 months.
- Regime 2 (*experimental treatment*): Continue taking PcP prophylaxis if HIV RNA > 400 copies/ml, and stop if the patient has confirmed viral suppression (two measurements of HIV RNA < 400).

The point of randomisation (“time 0”) was defined to be the visit at which a patient first met the eligibility criteria. There were 4813 patients complying with eligibility conditions, followed-up for a maximum of 5 years within the period 1998 to 2015. The analysis of the original study indicated that the risk of PcP diagnosis is approximately the same when PcP prophylaxis is stopped if patients are virally suppressed [8]. For illustrative purposes, we focus on a secondary endpoint from the original study, the time to PcP diagnosis or death (from any cause).

### The estimand for the emulated trial

Adopting the vocabulary of the ICH E9 addendum for time to event endpoints [9], we define the target study estimand in terms of the:

- Target population: PLWHA taking PcP prophylaxis;
- Treatment regimes: Stopping PcP prophylaxis according to (1) CD4 count or (2) viral suppression.
- Outcome: the time from randomization (*time 0*) until the person contracts Pneumocystis pneumonia (PcP) or dies (from any cause);
- Population-level summary measure: the hazard ratio comparing the difference between the two arms over the 5 year whole study period;
- Intercurrent event (ICE): “Non-compliance with treatment regime”. (Of note, people can be consistent with either treatment regime even when they stop prophylaxis). This ICE was handled using an “on treatment strategy” (perhaps better here “on regime” to avoid confusion with PcP prophylaxis treatment).

### Primary analysis of the estimand

By applying the eligibility criteria defined for the study to the COHERE observational database, all selected patients are consistent with *both* treatment regimes at the start of the emulated trial. Therefore, every patient was duplicated (cloned), and assigned to both treatment regimes at time 0. All subsequent visits for these patients were included in the analysis until the event of interest (PcP diagnosis/death), loss to follow-up (LTFU), non-compliance with the randomised regime (the relevant ICE), or the maximum period of study of 5 years was reached.

As primary analysis, a pooled logistic regression model was fitted to estimate the hazard ratio comparing the two regimes. To adjust for potential selection bias from the censoring process due to non-compliance with the randomized treatment regime (the ICE), inverse probability weights (IPWs) per patient month were included in the model (details in Appendix A and [8]). By fitting the pooled logistic model *including* the weights, we heuristically abrogate the effect of censoring due to the ICE, effectively creating a pseudo-population in which people followed their randomized regime—in line with our on treatment strategy. This establishes the rationale for the primary analysis providing estimates which provide statistically valid inference concerning the estimand, albeit under the assumptions that (1) there is no unmeasured confounding, and (2) censoring for any reason (including the ICE) is at random. In this context, censoring may be considered as a type of missing data process, and we adapt the well-known framework proposed by Little and Rubin [10]: a censoring at random (CAR) mechanism implies that the censoring and event time distribution are independent, conditional on the observed outcome and covariates; whereas censoring not at random (CNAR, also known as *informative* censoring) implies that the censoring and event processes are dependent, even after conditioning on the observed data.

The overall analysis approach is often known as the “clone-censor-weight” methodology for emulation trials from observational data [11, 12]

### Sensitivity analysis of the estimand

In the context of studies involving PLWHA, those censored due to being lost to follow-up are often considered to be higher at risk than those completing a study—so our approach now considers potential informative censoring scenarios for these people in the context of a sensitivity analysis.

We use a reference based multiple imputation (RBMI) approach to perform the sensitivity analysis [12]. To illustrate the general approach, we make the assumption that patients lost to follow-up on either regime stop taking PcP prophylaxis at the time they are lost to follow-up. Given the clinical context, this seems reasonable, but this is just one of potentially many scenarios that might be considered. To implement this, as imputation model we fit the pooled logistic regression model of the primary analysis to the subset of eligible patients that stopped prophylaxis. Once we have fitted the imputation model to this group of patients, we multiply impute new event times for those LTFU (refer to Appendix B for details). In this way, we have multiply imputed LTFU patients *with reference to* the hazard of patients off prophylaxis; that is, post-LTFU they have “jumped to off prophylaxis”.

We acknowledge that making the assumption that people stop taking prophylaxis at the exact time point when they are LTFU is conservative—they may stop later (or not at all). Also, by stopping prophylaxis they may no longer be adherent with their assigned regime and should be censored at this ICE. Therefore, our sensitivity analysis is perhaps better characterised as a worst-case situation in which those LTFU stop taking prophylaxis at, or sometime after, they are LTFU.

## Results

We compared the hazard ratio for the difference between the two treatment regimes from the primary analysis (including inverse probability weights), which assumed censoring was at random, with the sensitivity analysis in which the lost to follow-up patients on both arms “jumped to off prophylaxis”.

The original fully adjusted, IP weighted primary analysis indicated a protective effect for the new regime using viral suppression as stopping criteria (hazard ratio (HR) 0.78, 95% confidence interval [0.69, 0.89], *p* < 0.001, Table 1). In an additional step, we also multiply imputed those LTFU under CAR (as a cross-check to the primary analysis), and obtained similar results (HR 0.79 [0.68, 0.91], *p* = 0.002), which is in line with our expectations. For the sensitivity analysis, when we apply the “jump to off prophylaxis” approach for those LTFU, the hazard ratio attenuates compared to that from the primary analysis (HR 0.81 [0.69, 0.95], *p* = 0.009). The sensitivity analysis confirmed that regime 2 exhibits a clear improvement over the existing guidelines for PcP prophylaxis even when those LTFU “jump to off prophylaxis”. We conclude that there is no discernible additional risk from adopting the new regime for stopping PcP prophylaxis, although non-inferiority cannot be inferred without a priori reframing the hypothetical target trial in these terms.

In a further *posthoc* step, we also modelled a situation in which only patients LTFU on the new regime stopped taking prophylaxis. In this asymmetric case, the hazard ratio was almost the same as for the primary analysis but with slightly wider confidence intervals (HR 0.78 [0.66, 0.93], *p* = 0.005).

## Discussion

In our study, we emulated a hypothetical randomised trial using inverse probability weighting to adjust for potential censoring selection bias which implicitly makes the censoring at random assumption. We investigated a specific sensitivity analysis in which those lost to follow-up were assumed to stop PcP prophylaxis at some point after they had ceased their follow-up visits, and such patients adopted the hazard from an appropriate reference patient group to multiply impute new event times. The resulting hazard ratio estimate from this sensitivity analysis was consistent with the results from the primary analysis. Our sensitivity analysis approach is similar to that discussed in Pham et al. in which treatment discontinuation after being lost to follow-up can also be imputed [13].

Whilst we have used the hazard ratio as the main population level measure of the study, this has known drawbacks in terms of its causal interpretation [14]. However, this should not detract from the generic applicability of RBMI for performing sensitivity analysis; we could equally well have fitted a parametric survival function to estimate the absolute risk difference, used a non-parametric approach, or adopted an area under the curve method such as the restricted mean survival time [15].

An alternative approach to sensitivity analysis in this context was proposed by Lodi and colleagues [7]. In the sensitivity analysis of their study, they assumed that deaths with unknown cause were assumed to all be non-AIDS related, and then repeated the analysis. However, they note that this may be rather extreme and unrealistic in the context of their study. Whilst simple to implement, an alternative approach with more granular options such as that provided by using RBMI might have been a better alternative for the sensitivity analysis.

Another approach to carrying out our sensitivity analysis would be to manipulate the inverse probability weights in some directed manner - but this has similar drawbacks to “delta-type” sensitivity analysis methods [16–18]: These methods can be difficult to verify in terms of their clinical plausibility since the adopted weight multiplier, especially on the log odds scale, is often less than intuitive to understand. Reference based multiple imputation, as implemented here, avoids discussion of an appropriate multiplying factor by exploring sensitivities based on the post-censoring behavior of clearly defined and understandable groups of patients. Our patients were assumed to stop taking prophylaxis when they were lost to follow-up, which is a not only simple to understand, but also clinically plausible.

Our approach is comparatively straightforward to implement requiring only a slight modification of standard multiple imputation techniques. It also avoids dimensioning a sensitivity analysis parameter (and potentially its distribution) [19], or fully modelling the missing data process, which is often a rather complex and time-consuming process requiring specialised statistical knowledge. This recommends reference-based methods in terms of both their practicality and clinical plausibility. However, as in most things “context is everything”: It is the responsibility of the stakeholders in a specific study to consider the appropriate hazard for each group of censored patients, and its timing.

RBMI does have some potential drawbacks. There is standard software for multiply imputing time to event data [20, 21], but this currently does not include RBMI approaches. Depending on the context, if it is possible to align the IPW multiplier used for the sensitivity analysis to a clear clinical context, then we acknowledge the relative simplicity of pursuing such an approach. Combining IPW and MI as demonstrated here requires a certain degree of statistical expertise, but most of the complexity concerns the definition of the target and emulated trials, and recognizing the differences between the two.

## Conclusion

Given the now prominent role of the estimand framework and sensitivity analyses in trials, not least exemplified by the ICH E9 addendum, it is important to provide methods which are not only easy to implement and use, but which are also clinically plausible and contextually relevant to the trial team and other stakeholders. As the application presented here demonstrates, reference based multiple imputation is a simple approach that can be used either in a primary analysis or for a sensitivity analysis in this context.

**Table 1:** Patient numbers at baseline and at the end of follow-up with hazard ratio estimates from the primary analysis using IPW, multiply imputed under CAR for those LTFU, along with the sensitivity analysis assuming those lost to follow-up “jumped to off prophylaxis”; hazard ratios < 1 indicate that the new regime 2 using viral suppression as criteria *reduces* risk compared to the existing strategy based on CD4 count

Time point		Regime 1 Existing prophylaxis guidelines	Regime 2 New prophylaxis strategy	*p*-value
Baseline (time 0)	Total patients	4813	4813	
Follow-up	Event: PcP diagnosis	52 (1.1%)	51 (1.1%)	0.9^*^
Follow-up	Event: Died	183 (3.8%)	158 (3.3%)	0.2^*^
Follow-up	Lost to follow-up (LTFU)	233 (4.8%)	216 (4.5%)	0.4^*^
Primary analysis	Event: PcP diagnosis/deathHR under CAR^#^ (using IPW) (HR [95% CI])	235 (9.4%) 1.0 (reference)	209 (5.5%) 0.78 [0.69, 0.89]	< 0.001
Supplementary analysis	HR under CAR^#^ (using IPW and MI for LTFU)	1.0 (reference)	0.79 [0.68, 0.91]	0.002
Sensitivity analysis 1	HR under *“jump to off prophylaxis”* for those LTFU^#^ (using IPW and MI)	1.0 (reference)	0.81 [0.69, 0.95]	0.009
Sensitivity analysis 2	HR under *“jump to off prophylaxis”* for those LTFU on regime 2 only^#^ (using IPW and MI)	1.0 (reference)	0.78 [0.66, 0.93]	0.005

*CI* confidence interval, CAR censoring at random; *IPW* inverse probability weighting;^*^p-value from a chi-square test comparing proportions for regimes 1 versus 2; ^#^Model has PcP diagnosis/death as dependent variable and the following independent variables: indicator variable for the regime along with baseline hazard (time, time^2^ and time^3^), and an interaction term between time and the regime

## Electronic supplementary material

Below is the link to the electronic supplementary material.

Supplementary Material 1

## Data Availability

Data used for the analysis will generally not be publicly available, but can be made available based on the approval by the chair of the executive committee of COHERE (Stephane De Wit; refer to author list).
