# Supplementary material for: Application of the estimand framework for an emulated trial using reference based multiple imputation to investigate informative censoring
Source: BMC Med Res Methodol. 2024 Oct 18;24:245. doi: 10.1186/s12874-024-02364-6 (PMC11487792; doi:10.1186/s12874-024-02364-6)
Supplement: Supplementary file 1 — Supplementary Material 1 [file 12874_2024_2364_MOESM1_ESM.pdf]

Supplementary material

Table S1: Baseline characteristics for patients in the emulated trial

	**Overall**	**No PcP diagnosis**	**PcP diagnosis**	**P-value**
Number of patients	4’813	4’761	52 (1.1%)	-
Female (%)	1195 (24.8%)	1’182 (24.8%)	13 (25.5%)	0.99
Age in years (median [IQR])	40 [35, 47]	40 [25, 47]	40 [33, 46]	0.58
Geographical orgin				0.54
Europe	3'938 (81.8%)	3'895 (81.8%)	43 (84.3%)	
Africa	482 (10.0%)	478 (10.0%)	4 (7.8%)	
Asia	83 (1.7%)	82 (1.7%)	1 (2.0%)	
Latin America	236 (4.9%)	235 (4.9%)	1 (2.0%)	
North Africa and Middle East	74 (1.5%)	72 (1.5%)	2 (3.9%)	
HIV transmission mode (%)				0.44
MSM	1’606 (33.4%)	1’586 (33.3%)	20 (39.2%)	
Heterosexual	1’833 (38.1%)	1’815 (38.1%)	18 (35.3%)	
IDU	1’155 (24.0%)	1’146 (24.1%)	9 (17.6%)	
Other	219 (4.6%)	215 (4.5%)	4 (7.8%)	
CD4 (cells/µL)	130 [77, 169]	130 [77, 169]	120 [53, 159]	0.11
HIV RNA (copies/ml)	1460 [107, 65000]	1402 [102, 63816]	46700 [540, 227600]	<0.001
Calendar year at start of emulated trial	2003 [1999, 2008]	2003 [1999, 2008]	2002 [1998, 2006]	0.18

% of follow-up on ART	84% [41%, 100%]	84% [41%, 100%]	100% [84%, 100%]	<0.001de

Appendix A: Details of the primary analysis model

To model the baseline hazard, we included *time* (months from *time 0*), along with its square and cubic terms. The model also included an indicator variable for the regime, along with an interaction term between *regime* and *time* to allow for non-proportional hazards.~~ The following baseline variables were included in the model; *gender*, *age*, *geographical origin* (Europe (reference), Africa, Asia, Latin America, North Africa and Middles East), *transmission mode* (Heterosexual (reference), MSM, IDU, Other), *CD4* (and its square), *HIV RNA* (and its square), *calendar year at time 0* for each patient (to take into account changes in guidelines), a variable defining the percentage of post-baseline (i.e. post-randomization) follow-up time on cART, and an indicator variable for those LTFU (not included when multiply imputing)~~.

We calculated inverse probability weights by fitting a logistic model with the indicator for censoring due to non-compliance with randomized regime~~treatment~~ (i.e. the ICE) as dependent variable, along with the~~ prophylaxis~~ regime, and baseline and time varying covariates as independent variables: *gender*, *age*, *geographical origin* (Europe (reference), Africa, Asia, Latin America, North Africa and Middles East), *transmission mode* (Heterosexual (reference), MSM, IDU, Other), *CD4* (and its square), *HIV RNA* (and its square), *calendar year at time 0* for each patient (to take into account changes in guidelines), a variable defining the percentage of post-baseline (i.e. post-randomization) follow-up time on cART, and an indicator variable for those LTFU (not included when multiply imputing).

~~. ~~To ensure the weights provided bounded estimates, we stabilized the weights and truncated them to avoid large value. Robust sandwich-type errors were calculated to account for intra-patient correlation since the analysis set includes multiple visits per patient.

Further details of the study and R code are available in the supplementary material of [8].

Appendix B: Multiple imputation

Censoring at random (CAR)

In the following, the inverse probability weights (IPWs) relating to censoring of patients that were no longer compliant with their respective regime, are included as for the primary analysis. For those people "lost to follow-up", we begin by multiply imputing new event times for such patients under the censoring at random (CAR) assumption; this is equivalent to a scenario in which people continue taking, respectively not taking, prophylaxis in line with their behavior at the time they were lost to follow-up.

The MI process fits a survival function to the observed data using a discrete time pooled logistic regression model, and uses this to generate new event times for patients LFTU. For example, we estimate a survival function for a specific patient LTFU, and we know that this patient was lost after, say, 12 months. We know that, based on the patient specific survival function, the probability of survival was, say 0.8, at 12 months. In order to impute a new event time (PcP diagnosis/death) for this patient, we generate a uniformly distributed variable on [0, 0.8], say 0.5. Based on the patient specific survival function, we estimate an event time corresponding to a survival probability of 0.5, say 3 years (note must be greater than the time LTFU occurred which was 12 months in this example). This is the imputed event time for this patient LTFU. If the imputed new event time was greater than 5 years, then since this after the study ended, we would assume the patient had no event within the study period. The patient survival function used in this instance is based on a set of estimates, say $\hat{\boldsymbol{\beta}}_{k=1}$. For the next imputed data set, we use a new set $\hat{\boldsymbol{\beta}}_{k=2}$ of estimates for the survival function, and proceed as above, for each patient LTFU.

The 3 main steps of the MI process assuming CAR for those LTFU are as follows:

Step 1: Define and fit the imputation model to the observed data

As imputation model, we fit a model predicting survival time based on all covariates we consider potentially relevant to the LTFU censoring process, along with those considered most probably not involved in the censoring mechanism, but nonetheless predictive of survival. We use this model to generate new event times for each patient

LTFU. As withIn line with the primary analysis, we fit an adjusted pooled logistic model to the observed data, with the associated survival function:

$$\hat{S}(t|\boldsymbol{A},\boldsymbol{W}) = \prod_{j:t_j \le J}[1 - (1 + \exp{(-\hat{\beta}_0(t_j) - \hat{\beta}_1\boldsymbol{A} - \hat{\boldsymbol{\beta}}_{..}\boldsymbol{W})})^{-1}],$$

for months j=1, 2, …, J, PcP treatment regime indicator **A**=1, 2, baseline and time varying covariates **W**, and the associated parameter estimates from the fitted model, β_0 (for the baseline hazard), β_1 (the effect for the treatment regime i.e. the primary endpoint), and finally, the vector of effects, $\hat{\boldsymbol{\beta}}_{..}$, pertaining to the design matrix **W**.

We assume the full set of parameter estimates from fitting this imputation model to the observed data (i.e. $\hat{\boldsymbol{\beta}} = (\hat{\beta}_0, \hat{\beta}_1, \hat{\boldsymbol{\beta}}_{..})$) are multivariate normally distributed. We then sample these estimates K times creating slightly different survival functions each time. These are used as the basis for generating the new event times for those censored, leading to K data sets.

Formally, we approximate the Bayesian posterior distribution by drawing K (e.g. 50) sets of estimates for the parameters from the asymptotic normal sampling distribution, $\mathcal{N}(\hat{\boldsymbol{\beta}},\ I(\hat{\boldsymbol{\beta}})^{-1})$, where the expected information is estimated by the observed sampling information (i.e. VAR($\hat{\boldsymbol{\beta}}$)). This results in K=50 sets of parameter estimates, $\hat{\boldsymbol{\beta}}_k$, k = 1,…, K.

Step 2: Generate K multiply imputed data sets

Using a specific set of parameters $\hat{\boldsymbol{\beta}}_k$ obtained from the imputation model in the survival function, we calculate the linear predictors, and use these to generate new event times for censored patients.

For each subject i, lost to follow-up at time T_i, we calculate

$$p_i = 1 - \hat{S}(t_i|\boldsymbol{A},\boldsymbol{W}),$$

where $\hat{S}$ is the survival function using the set of parameters $\hat{\boldsymbol{\beta}}_k$.

Using this p_i, the conditional survival probability for patient i at the time point when LTFU occurs, we draw a new value

$$u_i \sim \text{uniform}[p_i, 1],$$

which is the basis for calculating the new event time (ie PcP diagnosis or death) as the solution of

$$u_i = 1\text{-}\hat{S}(t_i|\boldsymbol{A},\boldsymbol{W}),$$

ensuring that the new event time is greater than the existing censoring time.

Now, the new event time for the LTFU patient j is generated by evaluating:

$$T_j^* = \frac{-\log(u_i)}{\prod_{j:t_j\le T}[1-(1+\exp(-\beta_0(t_j)-\beta_1\boldsymbol{A}-\boldsymbol{\beta}\boldsymbol{W}))^{-1}]}$$

from the imputation model.

This is a discrete time model based on post-baseline months, and so the expression above is a step function for the survival probability per month. We can generate a new event time (i.e. month of PcP diagnosis or death) by using a simple reverse look up of the time T_j^* corresponding to the "future" survival probability of the new event u_i. If the generated time is greater than 5 years, (the study period) then we assume that no event occurred within the study period, and designate it as (administratively) censored at 5 years.

We repeat the process for each patient LTFU in data set *k* to produce a single imputed data set. This process is then repeated for each of the other (*K-1*) (i.e. 49) sets of parameters $\hat{\boldsymbol{\beta}}_k$ in turn.

Step 3: Fit the analysis model

We then fit the same model we used for the primary analysis (including the IPWs), to each of the multiply imputed data sets in turn. This results in *K*=50 sets of parameter estimates, which we then average in the usual way using "Rubin's rules" [10].

This concludes the multiple imputation procedure assuming CAR, and constitutes the basis for investigating other scenarios in which the censoring process is informative. R code is provided below.

Censoring not at random

Step 1a: "jump to off prophylaxis"

For the sensitivity analysis, as imputation model we use the same model as that defined in Step 1 above, but this time fit the model only to the subset of patients that are not taking prophylaxis. This provides the estimated hazard for this subgroup, and we use this model to generate new event times for those LTFU on both regimes as above.

Code for multiple imputation under CAR and sensitivity analysis

```
#-----------------------------------------------------------------------------------------------------------
# FUNCTIONS

#-----------------------------------------------------------------------------------------------------
# survival probability at time t

# imputation model for CAR

pred.S<-function(pat, k, t) # calculates the linear predictor
{
  tempo<-unname(sum(c(imp.coef[k,1]
                    , ifelse(pat$rep==1, 0, imp.coef[k,2])  # this line deleted for the sensitivity analysis
                    , imp.coef[k,3]*t
                    , imp.coef[k,4]*t^2
                    , imp.coef[k,5]*t^3
                    , ifelse(pat$gender=="M", 0, imp.coef[k,6])
                    , ifelse(pat$mode2=="Heterosexual", 0,
                           ifelse(pat$mode2=="MSM", imp.coef[k,7],
                                 ifelse(pat$mode2=="IDU", imp.coef[k,8], imp.coef[k,9])))
                    , ifelse(pat$origin=="Europe", 0,
                           ifelse(pat$origin=="Africa", imp.coef[k,10],
                                 ifelse(pat$origin=="Asia", imp.coef[k,11],
                                   ifelse(pat$origin=="Latin America", imp.coef[k,12], imp.coef[k,13]))))
                    , imp.coef[k,14]*pat$sbcd4
                    , imp.coef[k,15]*pat$sbcd4^2
                    , imp.coef[k,16]*pat$log10brna
                    , imp.coef[k,17]*pat$log10brna^2
                    , imp.coef[k,18]*pat$pc_timeoncart
                    , imp.coef[k,19]*pat$YRbase
```

```
                    , imp.coef[k,20]*pat$age
                    , imp.coef[k,21]*pat$age^2
                    , imp.coef[k,22]*pat$scd4
                    , imp.coef[k,23]*pat$scd4^2
                    , imp.coef[k,24]*pat$log10rna
                    , imp.coef[k,25]*pat$log10rna^2
  )))
  return(tempo)
}

# scientific model - same as for primary analysis
mod=formula(EVENT_MI~      # multiply imputed pcp diagnosis or death
            as.factor(rep) # regime 1 or 2
            +newtime       # including new imputed times
            +I(newtime^2)
            +I(newtime^3)
            +as.factor(rep):time
            +bage
            +I(bage^2)
            +factor(gender)
            +factor(mode2)
            +factor(origin3)
            +sbcd4
            +I(sbcd4^2)
            +log10brna
            +I(log10brna^2)
            +pc_timeoncart
            +YRbase
)

#-----------------------------------------------------------------------------------------------
# initiate parameters
```

```
set.seed(12353)
K<-50
max_study<-60 # 5 yrs

for(k in i:K)
{
 for (i in 1:length(temp$patient))
 {
  pat<-temp[i,]

  if(temp$imp_ind[i]==1) # patient is censored
  {
   # define the X matrix for this censored subject
   # calculate S based on the lp, varying t
   t<-seq(1, max_study)
   # calculate the lp
   lp<-sapply(1:max_study, function(i) pred.S(pat, k, t[i])) # for varying time
   # Estimate of the hazard
   hall<-1/(1+exp(-lp))
   # Calculate survival probability censoring time
   Sall<-cumprod(1-hall)

   # sort S to find the interval using findInterval
   d<-data.frame(t, Sall

   # calculate S at the censoring time
   S<-Sall[pat$time]

   # Generate Uniform[p.i,1] variables only for the censored patients
   # This ensures that each imputed survival time is greater than the time at which
   # the unit was censored.
   p<-1-S
```

```
      U<-runif(1, p, 1)


      # invert S to find T*
      minS<-min(Sall)
      # sort data frame to use findInterval
      d<-d[with(d, order(-t)), ]


      if((1-U)<=minS)
      {
        newtime[[i]]=max_study #Time > longest study time - assume CAR & maximum study time
        R[[i]]=0
      }else{
        newtime[[i]]<-max(d[findInterval((1-U), d$S),1]-1, 0)
        tempo<-newtime[[i]]
        R[[i]]=1
      }
    }else{ # patient not censored
      newtime[[i]]=pat$time #existing time
      R[[i]]=0
    }
    #print(i)
  } # end of i loop
} # end of k loop
```
